# Synthesis of Poly(methyl methacrylate-co-butyl acrylate)/Perfluorosilyl Methacrylate Core-Shell Nanoparticles: Novel Approach for Optimization of Coating Process

**DOI:** 10.3390/polym10111186

**Published:** 2018-10-24

**Authors:** Jun-Won Kook, Yongsoo Kim, Kiseob Hwang, Jung Hyun Kim, Jun-Young Lee

**Affiliations:** 1 Korea Institute of Industrial Technology, 89 Yangdaegiro-gil, Ipjang-myeon, Seobuk-gu, Cheonan, Chungcheongnam-do 31056, Korea; kukjw83@kitech.re.kr (J.-W.K.); bohemian4215@kitech.re.kr (Y.K.); ks_hwang@kitech.re.kr (K.H.); 2 Department of Chemical and Biomolecular Engineering, Yonsei University, 50 Yonsei-ro, Seodamoon-gu, Seoul 03722, Korea; jayhkim@yonsei.ac.kr

**Keywords:** core-shell nanoparticles, optimization, surface properties, perfluorosilyl methacrylate

## Abstract

In this study, the coating order of two monomers in the shell polymerization process of core-shell nanoparticles was altered to facilitate easy coating and optimize the properties of the coated surface to simplify the additional coating formulation process. To obtain a glass transition temperature suitable for coating, a core was synthesized by the copolymerization of an acryl monomer. A perfluoro monomer and silane monomer were additionally added to synthesize nanoparticles exhibiting both water–oil repellency and anchoring properties. In order to realize various surface properties, the nanoparticles underwent surface modification and cellulose fiber was introduced. Through the various data described in this text, the surface properties improved with the order of the introduction of the two monomers.

## 1. Introduction

Fluorinated acrylate emulsions have attracted increasing research attention as the various films fabricated from these nanoparticles not only maintain their original properties such as good matrix adhesion [1], but also exhibit unique properties such as high thermal and chemical resistance [2,3,4], water repellency, good oxidation resistance, and strong fluorine electronegativity. However, they are limited in some applications; for example, the fluorinated films exhibit a poor anti-fouling property in seawater because when perfluoroalkyl-containing polymers make contact with water, the perfluoroalkyl groups on the surface migrate toward the polymer, reducing their interfacial free energy, while the non-fluorinated groups migrate toward the interface. This will cause the copolymers to possess surface free energy [5,6,7,8]. In addition, some products of poly(perfluoroalkyl acrylate) with long perfluoroalkyl groups (more than seven carbons) such as perfluorooctanoic acid and perfluorooctanesulfonic acid (PFOS) have been found to resist degradation [9,10]. Therefore, short perfluoroalkyl groups with seven or fewer carbon atoms play an important role in practical applications as studies involving non-human primates and human tissues have indicated that short perfluoroalkyl groups have substantially shorter half-lives and are less toxic than long perfluoroalkyl groups [11].

Currently, fluorinated acrylate polymers with various structures such as random [12] and hybrid nanocomposites [13], grafts, and core-shell nanoparticles [14,15,16] are synthesized by emulsion polymerization, suspension-emulsion-combined polymerization, seeded emulsion polymerization, and other synthetic polymerization methods. A core-shell fluorinated silyl methacrylate latex prepared via semi-continuous seeded emulsion polymerization is particularly attractive due to its advantage in film formation when compared to general emulsion, especially the latex, which consists of a fluorine-free acrylate core and fluorine-containing acrylic shell. During the film-forming process, the fluorine-containing acrylic shell preferentially migrates toward the surface and provides the materials with excellent surface properties.

However, some distinct drawbacks in conventional fluorinated acrylate latex may restrict the utilization of the particular advantages of fluorinated acrylate copolymer emulsions, e.g., residue emulsifiers in conventional emulsion, which can easily migrate to the interface between the polymer and solid substrate, leading to poor adhesion of the polymers on the substrates, while the fluoroalkyl groups migrate toward the inside of the film when placed in water, causing the latex film to perform poorly. It is thus imperative to develop new methods for preparing fluorinated acrylate copolymer emulsions and introduce new functional monomers to improve the properties of the emulsion and its latex film. To address the issue above-mentioned, an increasing number of studies have been conducted on the soap-free emulsion polymerization method [17,18,19,20,21,22,23,24,25,26] using reactive emulsifiers as the reactive emulsifier can react with monomers and become part of the polymers, decreasing the negative effects of general emulsifiers on the nature of the latex film. In addition, self-crosslinking functional monomers, which can copolymerize with other vinyl monomers to form fluorinated acrylate copolymer latexes, but also form a reticular polymer matrix during the film-forming process to restrict the fluoroalkyl groups from migrating inside, have gradually attracted increasing interest. 

Thus, the purpose of this study can be divided into two major categories. First, to control the content of the monomer with low and high glass transition temperatures, the core was polymerized so that it could easily be coated at the optimum temperature on the desired substrate. Second, 1*H*,1*H*,2*H*,2*H*-tridecafluoro-*n*-octyl acrylate (TFOA) and trimethoxysilyl propyl methacrylate (TPM) were injected sequentially or mixed in the shell polymerization process to reduce the additional coating formulation process. As a result, the roughness of the coated surface was uniform when the two monomers were added sequentially. 

It is believed that this method can be applied to conditions where the coating is limited by simplifying the coating formula using the silane coupling agent at the shell interface during the core-shell nanoparticle synthesis process. 

## 2. Materials and Methods

### 2.1. Materials

Methyl methacrylate (MMA), butyl acrylate (BA), 4-styrenesulfonic acid sodium salt hydrate (NaSS), ammonium persulfate (APS), and TPM were purchased from Sigma-Aldrich (St. Louis, MO, USA). TFOA was purchased from Tokyo Chemical Industry Co., (Tokyo, Japan). Cellulose fiber (CF, FIF 400 grade) was purchased from Nycon Materials Co., Ltd. (Asan, Korea). All chemicals were used as received without further purification. Deionized (DI) water was used in all experiments.

### 2.2. Synthesis of Poly(methyl methacrylate-co-butyl acrylate) (PMBA) Nanoparticles

The PMBA nanoparticles were prepared in a 300-mL double-jacketed glass reactor equipped with a mechanical stirrer and three inlets. First, NaSS (0.25 g, 1.12 mmol) was dissolved in 100-mL DI water. Next, MMA (6 g, 59.9 mmol) and BA (4 g, 31.2 mmol) were added into the reactor. Then, after APS (0.2 g, 1.1 mmol) was dissolved in 5-mL DI water, APS solution was slowly added into the reactor at a rate of 2.6 mL/h for 2 h. The combined solutions were stirred at 70 °C. 

### 2.3. Synthesis of PMBA/Perfluorosilyl Methacrylate (PMBA-PFSM) Core-Shell Nanoparticles

In the previously synthesized P(MMA-*co*-BA) nanoparticles, we synthesized P(MMA-*co*-BA) PFSM core-shell nanoparticles by mixing or sequentially injecting TPM and TFOA into the reactor. Here, the TPM (1.35 g, 5.4 mmol), TFOA (2 g, 4.8 mmol), and mixed monomer (TPM + TFOA [3.35 g]) were slowly added into the reactor at a rate of 1.56, 0.705, and 1.133 mL/h for 2 h, respectively. Then, the resultant mixture was stirred for 3 h at 300 rpm. 

### 2.4. Preparation of PMBA-PFSM Cellulose Fiber Nanocomposite by Surface Modification

To confirm its applicability as a paper coating material, the PBMA-PFSM cellulose fiber nanocomposite was prepared by surface modification of the synthesized PMBA-PFSM core-shell nanoparticles via reaction with cellulose fiber, as per the following process. First, the ratio of EtOH to DI-water (*v/v*) was adjusted to 8:2 (31.56 g:10 g) and the solution pH was adjusted to 5 using acetic acid. Then, 0.2 g of the dried core-shell nanoparticles were added and stirred for 24 h. 

### 2.5. Characterization

PMBA/PFSM core-shell nanoparticle morphologies were observed by field-emission scanning electron microscopy (FE-SEM; JSM 7001F, JEOL, Tokyo, Japan) and transmission electron microscopy (TEM; JEM-F200, JEOL, Tokyo, Japan). The samples for FE-SEM and TEM analysis were prepared by mounting diluted samples onto a silicon wafer and copper grids, respectively. An energy-dispersed X-ray spectroscopy (EDS) analyzer attached to the TEM instrument operated in the STEM mode was used to analyze the chemical compositions of the synthesized nanoparticles. FT-IR analysis was performed within a wavenumber range of 600–4000 cm^−1^ at room temperature using a PerkinElmer Spectrum 100 (PerkinElmer, Waltham, MA, USA). Solid-state NMR experiments were acquired with a Bruker Avance II^+^ 400 MHz NMR system (in Korea Basic Science Institute [KBSI] Seoul Western Center, Seoul, Koera). The thermal properties of these samples were measured by differential scanning calorimetry and thermogravimetric analysis (TGA) under a N_2_ atmosphere. The heating and cooling ranges were −100–100 °C at a rate of 10 °C/min. The heating range was from 30 to 800 °C at a rate of 10 °C/min. The static and dynamic contact angles were measured using a drop shape analyzer. 

The surface chemical composition of the PMBA/PFSM nanocomposite coatings was studied using an XPS instrument (K-alpha, Thermo Scientific, VG, UK). XPS spectra were recorded with a 400-µm spot size and monochromatic Al X-ray sources (1486.6 eV). The X-ray source was operated at a power of 250 W and the high voltage was maintained at 140 kV with a take-off angle of 90°. All spectra were calibrated by the C1s peak of the C–C bond at 285 eV. After that, different functional groups were assigned using the reported C1s chemical shifts. 

The surface topography of the films was investigated by atomic force microscopy (AFM, JPK Instruments, Berlin, Germany) in tapping mode with a scan size of 10 µm × 10 µm at 25 °C.

## 3. Results and Discussion

### 3.1. Effects of Change of Input Order on Fluorinated Monomer (TFOA) and Comonomer (TPM)

**Figure 1:**
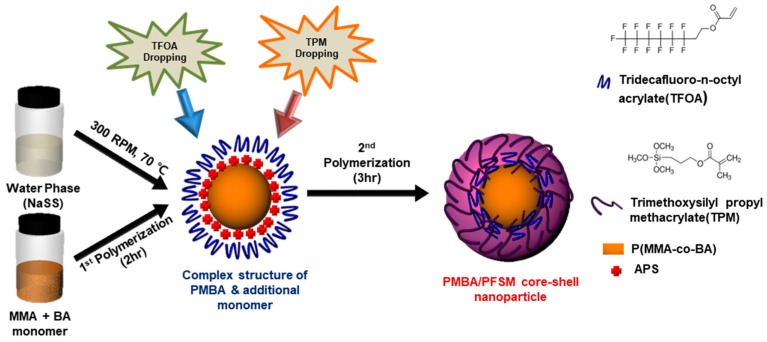
Schematic diagram of the PMBA/PFSM core-shell nanoparticle using the delay addition method.

Figure 1 shows a schematic of the PBMA/PFSM core-shell nanoparticles prepared by polymerizing the core nanoparticles with the MMA and BA monomer, followed by TFOA and TPM in a second shot using the delayed addition method. First, the reason for using a NaSS is that anionic surfactants are generally used in the emulsion polymerization method, which can inhibit the coating properties. Therefore, in this study, the surfactant directly participated in the reaction, and a reactive surfactant, NaSS, was used to improve the coating properties. In other words, it could easily migrate to the interface between the polymer and solid substrate, leading to poor adhesion of the polymer on the substrate. Second, to optimize the coating conditions at room temperature, when the core was polymerized, the weight ratio of MMA and BA was set to 1.5:1 (6.6 g:4.4 g) because the glass transition temperatures of poly(methyl methacrylate) and poly(butyl acrylate) are 105 and −49 °C, respectively. Here, if the core is polymerized with one of the two polymers, the desired morphology cannot be obtained at room temperature (RT) and the structure collapses. Therefore, the ratio of MMA and BA was set to 1.5:1, and the core was copolymerized to form spherical nanoparticles at RT. When the core was polymerized for 2 h, a conversion of about 80% was achieved, while the remaining 20% of the MMA and BA monomers existed at the core interface. When the conversion was 80%, we put in shell material as the MMA and BA residues in the continuous phase possess double bonds, which react with each other at the interface between TFOA and TPM to form a stable shell, and the polymerization time of the core was set at 2 h. In this case, TFOA and TPM were mixed or sequentially injected to form a core-shell structure by reacting double bonds with the BA and MMA monomers present at the core interface. Here, when TFOA and TPM were mixed, it was difficult to prove the optimization of the surface properties of the PMBA/PFSM core-shell nanoparticles because of their random arrangement at the core interface. In addition, there may be uncertainty as to whether shell parts were formed in the core-shell morphology. In contrast, when TFOA and TPM were sequentially added, TFOA was present primarily in the shell, and were independently present in the shell part to ensure that the characteristics could clearly be confirmed. PMBA/PFSM core-shell nanoparticles were synthesized by sequentially injecting TFOA and TPM to realize easy coating with the substrate and a higher contact angle. In other words, the probability of the presence of TPM on the surface of the core-shell nanoparticles increased, making the coating easier.

### 3.2. Chemical Structure of PMBA/PFSM Core-Shell Nanoparticles

**Figure 2:**
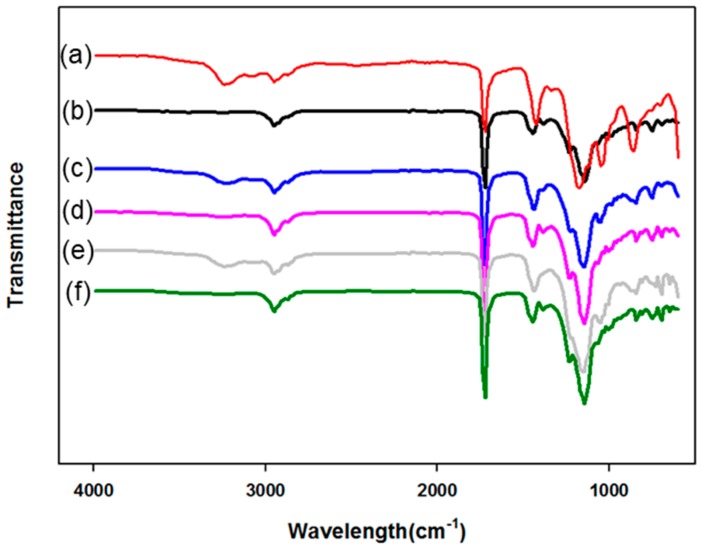
FT-IR spectra of the PMBA/PFSM core/shell nanoparticles by the change of input order and ratio of shell materials: (**a**) TCR-1, (**b**) TCR-2, (**c**) TCR-3, (**d**) TCR-4, (**e**) TCR-5, (**f**) TCR-6.

Figure 2 presents the FT-IR spectra of the PMBA/PFSM core-shell nanoparticles with a change in the input order and ratio of the shell materials. Let Figure 2a,c,e be “A”, which are the samples prepared by mixing TFOA and TPM, and Figure 2b,d,f be “B”, which are the samples prepared by sequentially injecting TFOA and TPM. In the spectrum, the peak at 1730 cm^−1^ is assigned to the stretching vibration of –C=O. The peaks at 1440 and 1388 cm^−1^ represent the symmetrical and asymmetrical vibration of –CH_3_, respectively. Moreover, after the core was polymerized, due to the formation by the reaction of unreacted acrylate monomers and TFOA at the core interface, the absorption peak at 1150 cm^−1^ may be the carboxyl group. Comparing A and B, we can see that the –OH stretching vibration represents A at 3250 cm^−1^ as TFOA and TPM were mixed and injected given the formation of –OH groups via the reaction between TFOA and TPM. 

**Figure 3:**
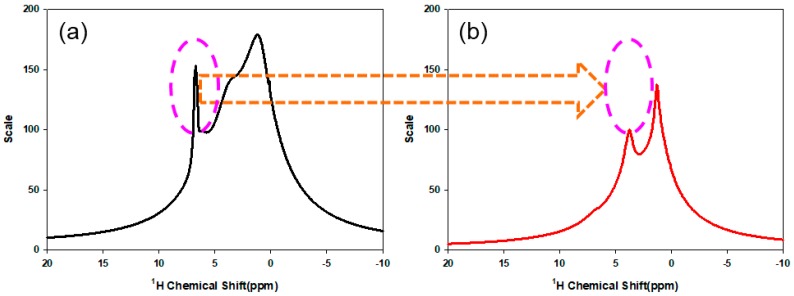
^1^H MAS NMR spectra of the TCR-1 sample. (**a**) Before hydrolysis, (**b**) After hydrolysis.

Figure 3a,b present a ^1^H MAS NMR spectrum of the TCR-1 sample for the confirmation of hydrolysis. Here, the purpose of confirming the hydrolysis of the TCR-1 sample was to react the CF through the surface modification of the TCR-1 sample to investigate the surface characteristics after coating. Comparing the results before and after hydrolysis, the peak in the range from 6 to 7 ppm in Figure 3a disappeared in Figure 3b, indicating hydrolysis. 

**Figure 4:**
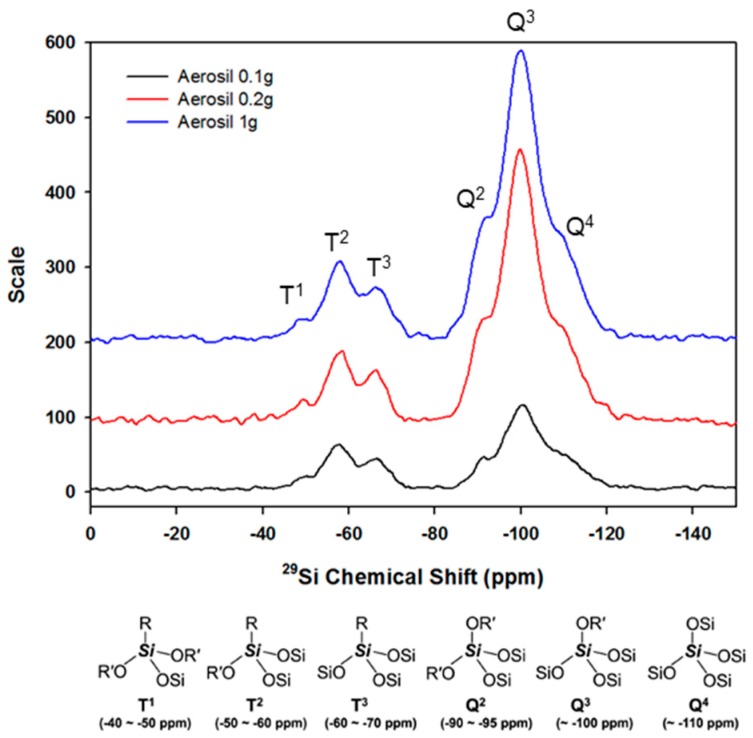
^29^Si CP/MAS NMR spectra for the TCR-1 sample by aerosol contents.

Next, Figure 4 presents the solid-state ^29^Si CP/MAS NMR analysis results, performed to determine whether the hydrolyzed TCR-1 sample reacted with the silane series (aerosol). Generally, the degree of reaction in ^29^Si CP/MAS NMR analysis can be seen from the peaks in the T and Q series groups. The related chemical structure is also displayed in Figure 4 [27]. Six signals were observed in the spectrum that can be assigned to different Si groups in the material. The Q^4^ group [Si(OSi)_4_] exhibited a resonance at about −110 ppm, while the Q^3^ group [Si(OSi)_3_OH] exhibited a resonance at −100 ppm. Additionally, a small resonance for the Q^2^ [Si(OSi)_2_OHR] groups was observed at −90 ppm. The T^3^ [Si(OSi)_3_R] and T^2^ [Si(OSi)_2_(OH)_2_] groups appeared at −59 and −50 ppm, respectively. Therefore, it can be confirmed that the hydrolyzed TCR-1 sample and aerosol reacted well.

### 3.3. Morphologies of PMBA/PFSM Core-Shell Nanoparticle and Nanocomposite

**Figure 5:**
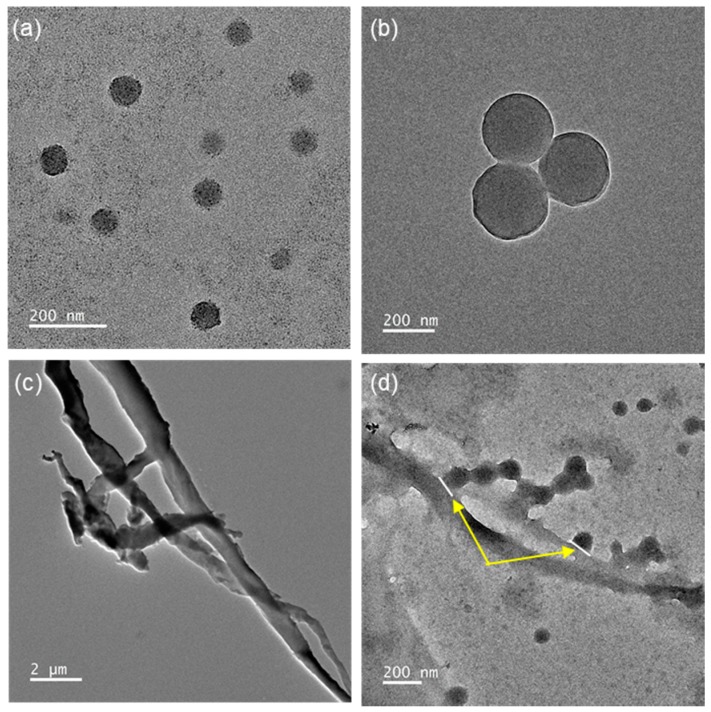
TEM images of the (**a**) TCR-1, (**b**) TCR-2, (**c**) only CF, and (**d**) TCR sample + CF nanocomposite reacting with hydrolysis CF.

Figure 5 shows the TEM images of the TCR-1, TCR-2, only CF, and TCR + CF nanocomposite samples. Figure 5a,b show that the average particle size was in the range of 100–200 nm. Figure 5b shows the core-shell structure clearly, whose structure was confirmed by the EDS mapping image below. The CF seed in Figure 5c had a diameter of 0.5–1 μm and appeared entangled. As shown in Figure 5d, the existence of the PMBA/PFSM nanoparticle at the CF interface indirectly indicates that the surface modification of the CF was successful.

**Figure 6:**
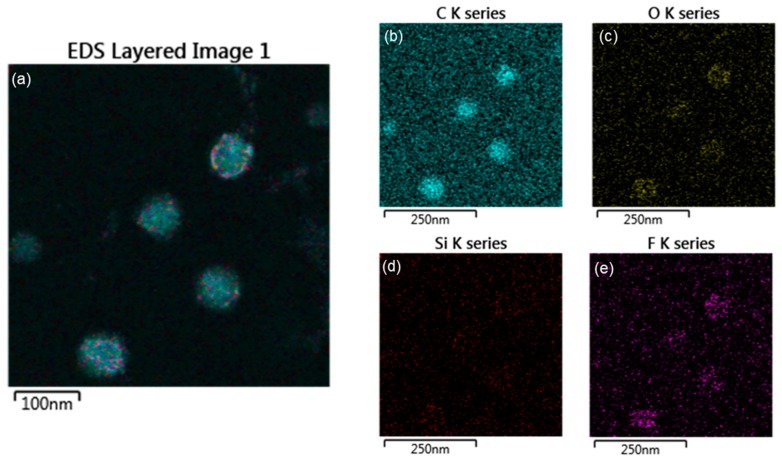
EDAX elemental mapping images of the TCR-1 sample. (**a**) EDS layered image, (**b**) C series, (**c**) O series, (**d**) Si series, and (**e**) F series (blue represents the C component, yellow represents the O component, red represents the Si component, and purple represents the F component in the mapping images).

**Figure 7:**
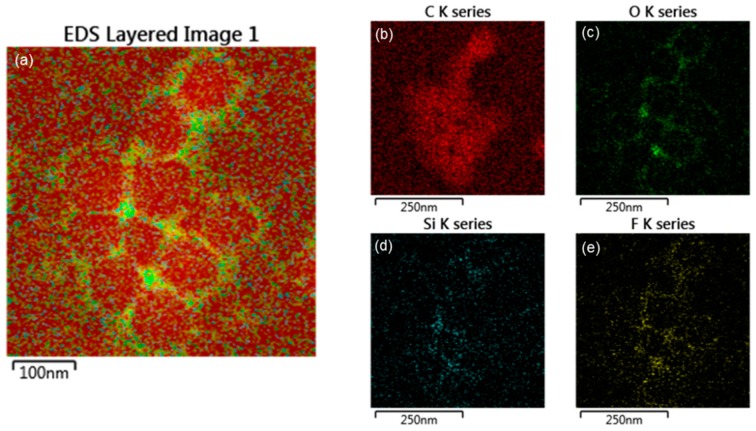
EDS elemental mapping images of the TCR-2 sample. (**a**) EDS layered image, (**b**) C series, (**c**) O series, (**d**) Si series, and (**e**) F series (red represents the C component, green represents the O component, blue represents the Si component, and yellow represents the F component in the mapping images).

To investigate the accuracy of the core-shell structure, EDS (electron mapping) analysis was performed, as shown in Figure 6 and Figure 7. The EDS mapping images show the distribution of each element in the core and shell of the nanoparticles. Figure 6 shows that in the TCR-1 sample produced by mixing TFOA and TPM together, the two monomers did not react properly at the core interface and were randomly located. On the other hand, Figure 7 shows that TFOA and TPM were sequentially injected during the synthesis of the TCR-2 sample, such that O, Si, and F series components were present in the shell part, while C series components were absent. Thus, the core-shell structure was elucidated using EDS mapping images when the TFOA and TPM were sequentially injected. 

### 3.4. Physical and Thermal Properties of PMBA/PFSM Core-Shell Nanoparticles

Table 1 presents the values of the particle size and contact angle (water/hexadecane [HD]) of the PMBA/PFSM core-shell nanoparticles according to the order and amount of the addition of TFOA and TPM. Let us set the TC-1, 3, and 5 sample groups to “A” and the TCR-2, 4, and 6 sample groups to “B”. The particle size and the contact angles in both water and HD increased with increasing TFOA content. This is because the core was first polymerized in the PMBA/PFSM core-shell nanoparticle and added for shell formation. With increasing TFOA content, the repulsive force exerted by the water and HD on the surface coated with the fluorinated group of TFOA increased, thus increasing the contact angles. 

**Figure 8:**
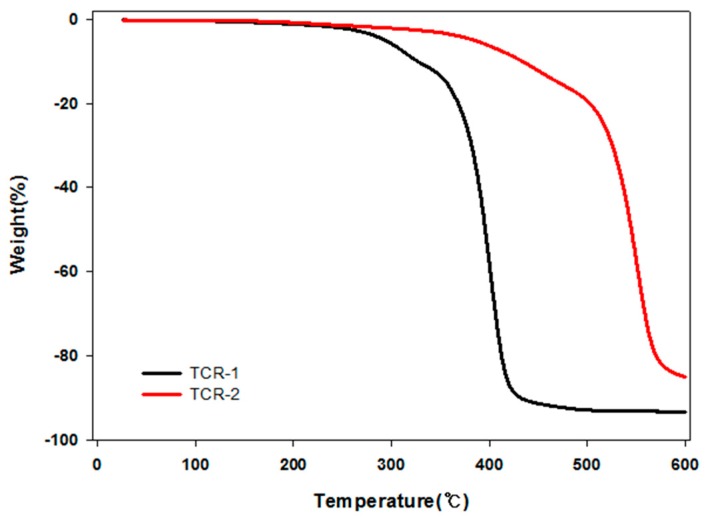
Thermal stability of the PMBA/PFSM core-shell nanoparticles to the order of injection.

In the TCR-A and B series, the content of TPM was controlled. At 20% and 30%, the measurement of the particle size was impossible with increasing viscosity. However, it was possible at 5% TPM and the highest values of the contact angle were 30% for TFOA and 5% for TPM. It was found that this condition was the optimum condition with the highest contact angle value. Next, the thermal stability of TFOA and TPM by injection order is illustrated in Figure 8 through TGA analysis. From the comparison of the pyrolysis start temperatures of TCR-1 and TCR-2, it can be seen that the temperature increased from about 400 to 550 °C. This is because the TCR-2 sample has a cross-linked density that completely reacts with the shell part of the core-shell nanoparticles by sequentially injecting TFOA and TPM.

### 3.5. Surface Chemical Composition of PMBA/PFSM Nanocomposite Coatings

**Figure 9:**
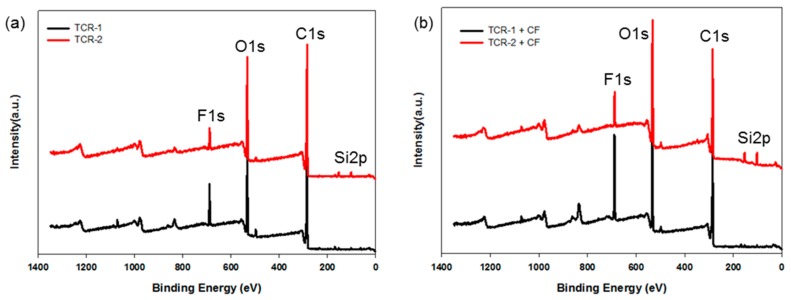
XPS survey spectra of the (**a**) TCR-1, TCR-2, (**b**) TCR + CF nanocomposites (TCR-1 + CF, TCR-2 + CF).

The chemical states of the TCR-1 and 2, and TCR + CF nanocomposite samples were investigated by XPS measurements, and the corresponding wide-scan spectra are plotted in Figure 9. For all samples, due to the PMBA/PFSM cellulose-based components, the C1s and O1s species were the dominant signals and occurred at binding energies (BEs) of 285.0 and 531.88 eV, respectively. Fluorine and silicon peaks were also found at about 688.0 and 105.0 eV. The chemical compositions of the TCR-1 and 2, and TCR + CF nanocomposite samples are listed in Table 2. The BEs of all the peaks were corrected relative to the reference peak (C–C, 285.0 eV). In Table 2, when comparing the TCR-1 and TCR-2 samples, there was almost no difference compared to the other elements, but the “Si” content increased. As shown in Figure 9b, compared to Figure 9a, both “O” and “Si” increased as TPM was present at the core-shell nanoparticle interface by sequentially injecting TFOA and TPM. In the case of the TCR sample and CF nanocomposite, the number of –OH bonds at the CF interface increased because of the surface modification of the particles.

**Figure 10:**
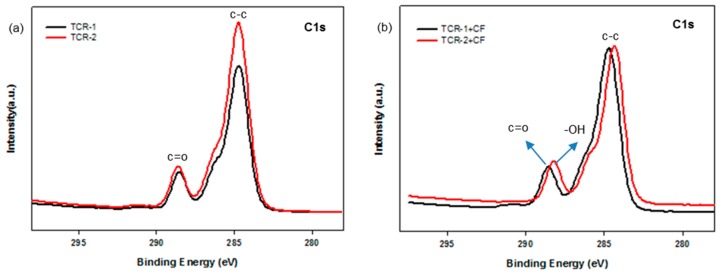
High-resolution XPS spectra with peak-fitting in the carbon BE region: (**a**) TCR-1, TCR-2, (**b**) TCR-1, 2, and CF nanocomposites.

Next, Figure 10 shows the fitted peaks in the region of the carbon BE of the TCR and TCR + CF nanocomposite sample. The C1s spectrum revealed the presence of two different types of carbon atoms (functional groups). Considering that the peak located at 285.0 eV represents aliphatic C–C/C–H, the other peak appearing at about 288.0 eV can be linked to carbon atoms belonging to –C=O-based functional groups (Figure 10a). Moreover, in Figure 10b, the peak at about 288.0 eV shifted to the right because the hydrolysis of the PBMA/PFSM nanoparticles resulted in the formation of –OH functional groups in the TCR + CF nanocomposite sample. 

### 3.6. Surface Topography of PMBA/PFSM Nanocomposite Coatings

**Figure 11:**
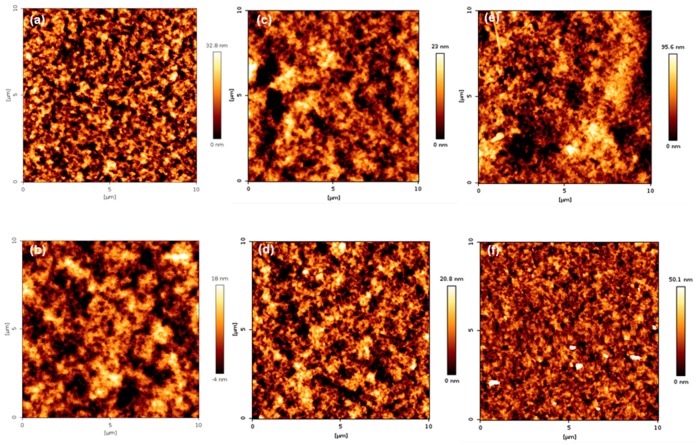
AFM height images of the TCR series samples: (**a**) TCR-1, (**b**) TCR-2, (**c**) TCR-3, (**d**) TCR-4, (**e**) TCR-5, and (**f**) TCR-6.

**Figure 12:**
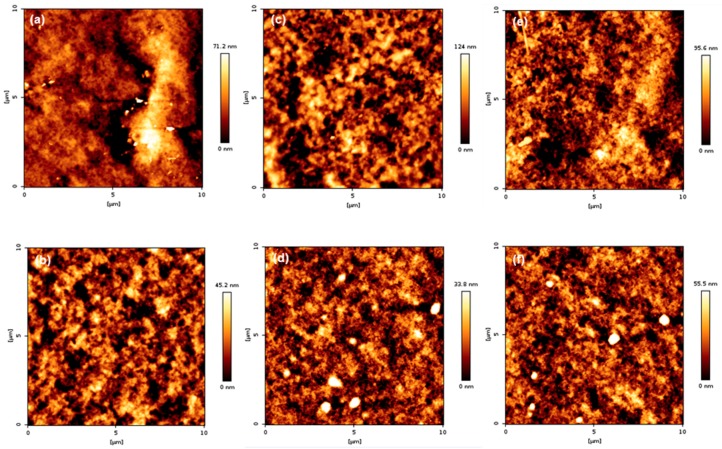
AFM height images of the TCR series + CF nanocomposite samples: (**a**) TCR-1 + CF, (**b**) TCR-2 + CF, (**c**) TCR-3 + CF, (**d**) TCR-4 + CF, (**e**) TCR-5 + CF, and (**f**) TCR-6 + CF.

The surface topography of the coatings is presented in Figure 11 and Figure 12. As shown in Figure 11, when comparing groups “A” and “B”, it can be seen that group B was more uniform than group A. In addition, the arithmetic average roughness (*R*_a_) and root mean square (*R*_q_) values were smaller than group A. The detailed values are listed in Table 3. The reason for the lower *R*_a_ and *R*_q_ values in group B is that TFOA and TPM were mixed in group A when the PBMA/PFSM coating solution was synthesized, and TFOA and TPM were sequentially introduced in group B. Therefore, the dispersion stability in group B increased, and the values of *R*_a_ and *R*_q_ were low. Figure 12 presents the AFM images of the sample obtained by reacting the CF with the hydrolyzed TCR sample. The overall behavior was similar to Figure 11, but the values of *R*_a_ and *R*_q_ were higher. Both *R*_a_ and *R*_q_ were calculated using Equations (1) and (2), respectively:

(1) $$R_{a}=\frac{1}{n}\sum_{i=1}^{n}\left|\gamma_{i}\right|,$$

(2) $$R_{q}=\sqrt{\frac{1}{n}\sum_{i=1}^{n}\gamma_{i}^{2}},$$

where *n* is the number of equally spaced measuring points along the trace and γ represents the vertical distance from the mean line. 

## 4. Conclusions

PMBA/PFSM core-shell nanoparticles were prepared by synthesizing PMBA nanoparticles as a core through delayed addition and then injecting TFOA and TPM. By the sequential addition of 30% TFOA and 5% TPM, the highest contact angle achieved was 142.4° and 101.4°, respectively, because if more than 5% of TPM was added, the viscosity of the PMBA/PFSM latex would increase and the particle shape would no longer be maintained. In addition, the thermal properties of the TFOA and TPM were investigated, and it was found that the pyrolysis temperature increased by about 150 °C.

To investigate the coated surface properties and the applicability to paper coating materials, solid-state ^29^Si NMR was analyzed to verify the reactivity of the prepared particles with CFs by surface modification of PMBA/PFSM core-shell nanoparticle. The TEM images and the EDS mapping images confirmed that the PMBA/PFSM core-shell nanoparticles had a defined core-shell structure, and it was found that CF reacted on the surface-modified nanoparticles. In addition, XPS analysis indicated that the PMBA/PFSM latex and PMBA/PFSM latex + CF nanocomposite were well coated on the substrate. Finally, the order of the addition of TFOA and TPM and the roughness of the CF nanocomposite sample were investigated. The *R*_a_ values of TFOA and TPM were found to decrease, while the CF nanocomposite sample exhibited identical behavior. 

In this approach, it was found that the thermal, physical, and surface characteristics of the PMBA/PFSM core-shell nanoparticle synthesis process were improved by sequentially injecting TFOA and TPM rather than mixing them together. In other words, we found that the order and content of TFOA and TPM constituting the shell material in the PMBA/PFSM core-shell nanoparticle synthesis process significantly influenced the optimization of the coating conditions. Furthermore, based on the data presented above, it is believed that the PMBA/PFSM core-shell nanoparticle can be fully utilized as a paper coating material. 

In this study, in order to check its applicability as a paper coating material, the nanocomposite was prepared by surface modification with nanoparticles by introducing cellulose fiber. Furthermore, it can be applied to various materials by confirming its applicability to hydrophobic substrates such as glass, steel sheet, and not paper.

## Figures and Tables

**Table 1:** The physical properties of PMBA/PFSM core-shell nanoparticles.

Sample	TFOA/TPM Weight Ratio (g)	Diameter (nm)	Contact Angle (°) [Water/HD]	PDI
TCR-1	2(15%)/1.35(10%)	123.7	130.5/72.8	0.201
TCR-2	2(15%)/1.35(10%)	130.1	135.1/74.9	0.123
TCR-3	2.7(20%)/1.35(10%)	186.3	138.6/77.6	0.103
TCR-4	2.7(20%)/1.35(10%)	190.1	135.1/76.3	0.006
TCR-5	4.87(30%)/1.35(10%)	270.5	135.1/92.1	0.101
TCR-6	4.87(30%)/1.35(10%)	256.3	142.4/101.4	0.088
TCR-A1	2.7(20%)/4.05(30%)	-	102.1/-	-
TCR-A2	2.7(20%)/2.7(20%)	-	106.2/-	-
TCR-A3	2.7(20%)/0.675(5%)	96.91	141.4/86.7	0.05
TCR-B1	4.87(30%)/4.05(30%)	-	107.1/-	-
TCR-B2	4.87(30%)/2.7(20%)	-	123.1/-	-
TCR-B3	4.87(30%)/0.675(5%)	106.3	140.3/97.9	0.034

**Table 2:** Chemical composition of the TCR-1, 2, and TCR + CF nanocomposite sample determined by XPS (atom %).

Sample	Concentration, % at wt			
	C1s	O1s	F1s	Si2p
**TCR-1**	73.37	23.06	2.59	0.99
**TCR-2**	73.19	21.75	2.67	2.38
**TCR-1+CF**	72.14	23.07	3.52	1.26
**TCR-2+CF**	62.72	29.45	1.35	6.49

**Table 3:** Calculated values of the arithmetic average roughness (*R*_a_) and the root mean squared roughness (*R*_q_) for the TCR series and TCR + CF nanocomposite sample.

**Roughness (nm)**	**TCR1**	**TCR2**	**TCR3**	**TCR4**	**TCR5**	**TCR6**
**R_a_**	5.93	4.01	4.01	3.75	17.17	6.86
**R_q_**	7.45	5.01	5.23	4.73	21.74	11.39
**Roughness (nm)**	**TCR1+CF**	**TCR2+CF**	**TCR3+CF**	**TCR4+CF**	**TCR5+CF**	**TCR6+CF**
**R_a_**	11.38	8.16	22.40	5.54	23.95	9.06
**R_q_**	16.19	10.28	28.22	7.68	31.73	12.62
